# Deep learning-based immunohistochemical estimation of breast cancer via ultrasound image applications

**DOI:** 10.3389/fonc.2023.1263685

**Published:** 2024-01-09

**Authors:** Ding Yan, Zijian Zhao, Jiajun Duan, Jia Qu, Linlin Shi, Qian Wang, Huawei Zhang

**Affiliations:** ^1^School of Control Science and Engineering, Shandong University, Jinan, China; ^2^School of Electrical Engineering and Telecommunications, University of New South Wales, Sydney, NSW, Australia; ^3^Department of Ultrasound, Shandong Provincial Hospital Affiliated to Shandong First Medical University, Jinan, China; ^4^Department of Ultrasound, Shandong Provincial Hospital, Cheeloo College of Medicine, Shandong University, Jinan, China

**Keywords:** computer-aided diagnosis, deep learning, neural network, immunohistochemistry, node of breast cancer

## Abstract

**Background:**

Breast cancer is the key global menace to women’s health, which ranks first by mortality rate. The rate reduction and early diagnostics of breast cancer are the mainstream of medical research. Immunohistochemical examination is the most important link in the process of breast cancer treatment, and its results directly affect physicians’ decision-making on follow-up medical treatment.

**Purpose:**

This study aims to develop a computer-aided diagnosis (CAD) method based on deep learning to classify breast ultrasound (BUS) images according to immunohistochemical results.

**Methods:**

A new depth learning framework guided by BUS image data analysis was proposed for the classification of breast cancer nodes in BUS images. The proposed CAD classification network mainly comprised three innovation points. First, a multilevel feature distillation network (MFD-Net) based on CNN, which could extract feature layers of different scales, was designed. Then, the image features extracted at different depths were fused to achieve multilevel feature distillation using depth separable convolution and reverse depth separable convolution to increase convolution depths. Finally, a new attention module containing two independent submodules, the channel attention module (CAM) and the spatial attention module (SAM), was introduced to improve the model classification ability in channel and space.

**Results:**

A total of 500 axial BUS images were retrieved from 294 patients who underwent BUS examination, and these images were detected and cropped, resulting in breast cancer node BUS image datasets, which were classified according to immunohistochemical findings, and the datasets were randomly subdivided into a training set (70%) and a test set (30%) in the classification process, with the results of the four immune indices output simultaneously from training and testing, in the model comparison experiment. Taking ER immune indicators as an example, the proposed model achieved a precision of 0.8933, a recall of 0.7563, an F1 score of 0.8191, and an accuracy of 0.8386, significantly outperforming the other models. The results of the designed ablation experiment also showed that the proposed multistage characteristic distillation structure and attention module were key in improving the accuracy rate.

**Conclusion:**

The extensive experiments verify the high efficiency of the proposed method. It is considered the first classification of breast cancer by immunohistochemical results in breast cancer image processing, and it provides an effective aid for postoperative breast cancer treatment, greatly reduces the difficulty of diagnosis for doctors, and improves work efficiency.

## 1 Introduction

Breast cancer is a malignant tumor of the mammary glands with the highest incidence rate among women. It is difficult to treat if found in advanced stages, but early detection can significantly increase survival and improve the lives of millions of women.

Breast ultrasound (BUS) is a widely adopted imaging modality for early breast cancer diagnosis and has the advantages of being non-invasive, safe, and relatively inexpensive; BUS can reduce the workload of radiologists and improve diagnostic accuracy (1). BUS examination is divided into categories; BUS is a common mode, which can show breast tomographic anatomy information and dynamically observe the dynamic changes of breast tissue structure over time in real time. However, in breast image acquisition and interpretation, the accuracy of ultrasonography is highly dependent on the skill and expertise of the radiologist (2). To overcome mistakes in judgment due to multiple causes, a computer-aided diagnostic (CAD) program was applied to BUS image processing. CAD is an image analysis procedure that enables the morphological analysis of breast lesions on BUS for effective detection and classification.

Immunohistochemistry is mainly based on the qualitative, localized, or quantitative detection of a cell’s corresponding antigen or antibody with a labeled antibody or antigen, observed with a microscope or electron microscope after a chemical chromogenic reaction. The microscopic morphological appearance of breast tissue has always been the basis of chemotherapeutic diagnosis by pathologists. Still, as the medical level progresses and public health claims continue to improve, the pathological specimens also progress toward a minimally invasive direction (3). Due to the heterogeneity of cancer tumor tissue, a large number of cancerous lymph nodes with different manifestations on different immune cells have emerged, posing a major challenge to diagnosis (4). For the mammary gland, which has a rich blood supply and lymphoid tissue distribution, the malignancy at this site may belong to the primary and may also metastasize from other sites. Pathologists are equally plagued by the search for microinvasion in carcinoma *in situ* or the presence of vascular tumor thrombus and perineural invasion in invasive carcinoma (5). Based on this, the application of immunohistochemical staining techniques has highlighted a great significance in the pathological diagnosis of breast cancer, and the commonly used immunological markers for breast cancer are P63, CK5/6, ER, PR, HER-2, P120, E-cad, EMA, MUC-1, EGFR, Ki-67, P53, and so on. Using these immunological markers, pathologists could provide better directions for further diagnosis and chemotherapy.

Recently, deep learning techniques, especially convolutional neural networks (CNNs) (6), have successfully solved different classification tasks using BUS examination in the CAD domain (7). Following this trend, Rakhlin et al. used several deep neural network structures and gradient enhancement tree classifiers to perform two and four classification tasks on breast cancer histological images (8). Alternatively, Vang et al. improved the multiclass breast cancer image classification sensitivity of the normal and benign predicted classes by designing a dual path network (DPN) to be used as a feature extractor (9). Golatkar et al. proposed a deep learning-based method for the classification of H&E-stained breast tissue images released for the BACH challenge 2018 by fine-tuning the Inception-v3 neural network (10). In 2019, Haarburger et al. proposed a 3D CNN and a multiscale curriculum learning strategy to classify malignancy globally based on an MRI of the whole breast (11). Independently, Park et al. passed the resulting representation to a hidden layer and then to a soft-max layer to obtain benign and malignant predictions for each breast image (12). Patil et al. managed to improve the interpretation of classification results by localizing microscopic histopathology breast cancer images (13). In 2020, Boumaraf et al. classified mammographic masses into four assessment categories using the CAD system with modified genetic feature selection, featuring the backpropagation neural network (BPN) (4). Alternatively, Kalafi et al. proposed a new framework for classifying breast cancer lesions using an attention module in modified VGG16 (14). In 2021, Li pretrained two neural networks with different structures and used the convolutional neural network to extract the characteristics of features automatically, fuse the features extracted from the two structures, and finally use the classifier to classify the fused feature (15). Mo et al. first predefined the regions of interest (ROIs) and then classified the lesion inside the ROIs; then, they used the so-called HoVer-Trans block to extract the inter- and intralayer spatial information horizontally and vertically (16). With the development of immunohistochemical technology, it becomes more and more involved in cancer classifications. Thus, Chen et al. proposed a new method for predicting the immunohistochemical index by using contrast-enhanced ultrasound for several minutes (17). More recently, Jiang et al. added an image classifier that utilized the same global image features to perform image classification (18). The above studies were focused on the binary and multiclass identification of benign and malignant breast cancer medical images. As immunohistochemistry techniques advance, the classification of breast cancer images progressively aligns with these developments; this paper mainly extracts breast cancer nodes through a target detection network and then classifies breast nodules into immunohistochemical categories.

This study attempts to make the CAD process more consistent with radiologists’ diagnostic considerations by introducing a novel deep learning framework. The main contributions of this study are as follows:

1) We constructed a multistage feature distillation network (MFD-Net) based on CNN; the network, initially created and applied to image classification, was based on the innovative concept of extracting image features at multiple levels, where feature layers of different scales were extracted for the classification of breast cancer nodes in the fine-grained domain. By increasing different convolution depths using the depthwise separable convolution and the reverse one, image features extracted at different depths were fused to achieve multilevel feature extraction, further improving the depth and performance of feature extraction. In the subsequent process of image classification, a significant improvement in accuracy was achieved.

2) We proposed a new attention module called ESCA attention block; the newly added attention module optimized the classification network in spatial and channel directions simultaneously. This allowed the network to focus on key information within the feature maps extracted at each layer, thereby improving the classification accuracy. Compared with other attention modules, this module had a greater capacity to enhance the performance of the classification network.

3) We created, annotated, organized, and used a breast cancer node dataset containing 500 node images for the experiments. Multiple scales of cancerous nodes were detected through the YOLOv7 target detection network; nodes were cropped in the target detection result map to extract the ROI from the node images. Then, according to the immunohistochemical results of these breast tissues, the breast node image ROIs were classified by four immune indicators [estrogen receptor (ER), progesterone receptor (PR), human epidermal growth factor receptor 2 (HER-2), and Ki-67] to form a multilabel classification dataset.

4) To the best of the authors’ knowledge, this study is the first to classify BUS image datasets based on immunohistochemistry results. Additionally, we have introduced a novel classification network for the first time and ultimately applied this proposed classification network to BUS image datasets. Extensive experiments have demonstrated that the proposed method outperforms other advanced methods in classifying multiple immunohistochemical indicators of breast cancer ultrasound images. This achievement can be instrumental in screening large-scale breast cancer diseases. Extensive experiments proved that the proposed method achieved superior performance than other advanced methods in classifying multiple immunohistochemical indicators of breast cancer ultrasound images, which can be instrumental in screening large-scale breast cancer diseases.

The rest of this paper is organized as follows. We initiated by outlining the processing of the BUS image dataset we established and its corresponding immunohistochemistry results in Section 2. Following that, we introduced the proposed methodology in Section 3. After that, we described the experiments and results and next provided a comparative discussion of our results in Section 4. Finally, the main conclusions and limitations of the proposed approach were drawn in the last section.

## 2 Materials

**Figure 1:**
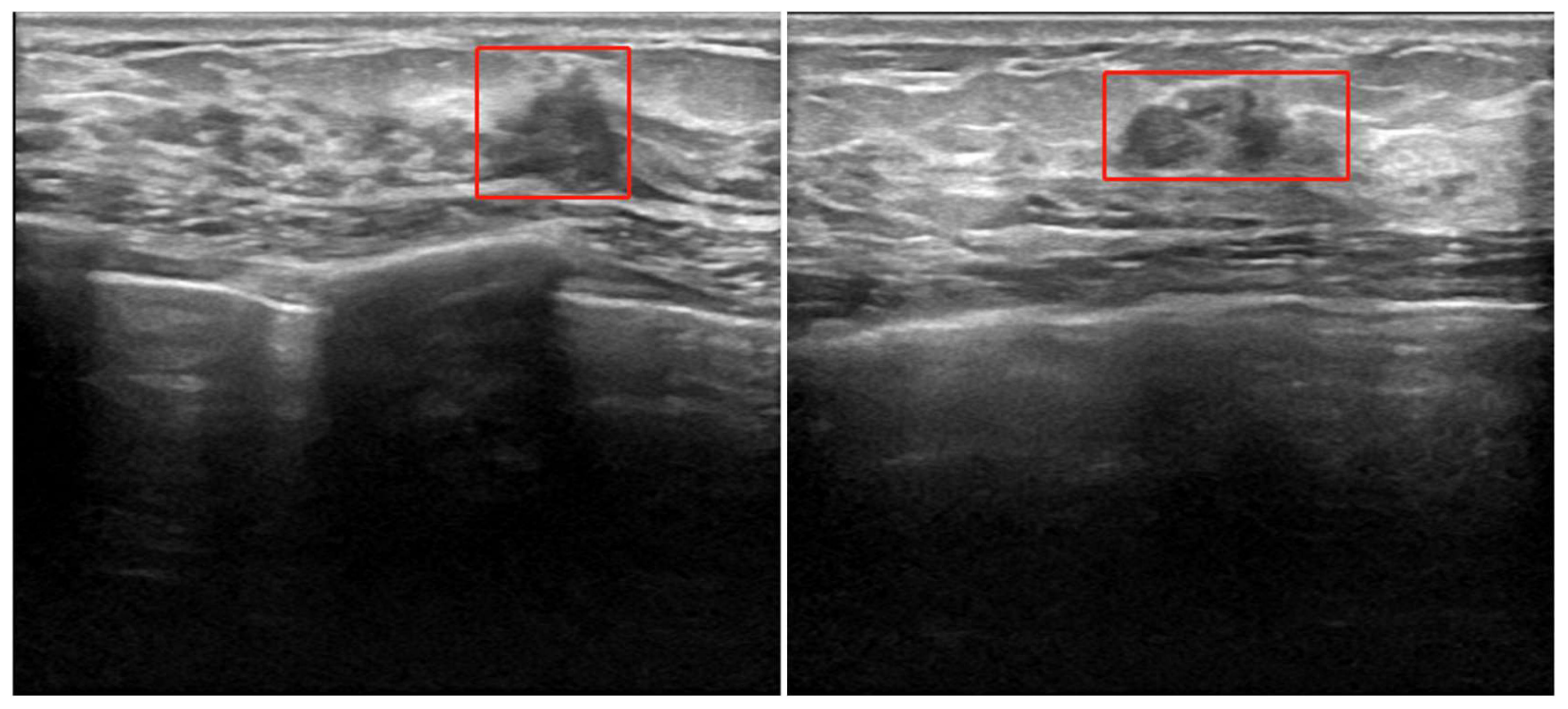
Experienced radiologists draw the ground truth ROI for cancer nodes.

The Ethics Committee of the Provincial Hospital Institutional Review Committee of Shandong First Medical University, China, approved the protocol of this retrospective study. The patients underwent ultrasonic and immunohistochemical examinations for surgical planning between January 2020 and May 2022. A total of 500 axial B ultrasound images were retrieved from 294 patients who underwent B ultrasound images for the assessment as “suspicious” breast cancer nodes in the earlier examinations. We have enhanced the dataset and expanded each ultrasound image to 20 and 500 BUS datasets to 10,000 using rotation, mirroring, brightness change, Gaussian noise, and other data enhancement technologies. Breast ultrasonography is the use of ultrasonic physical signals to diagnose breast diseases; ultrasound is delivered through the probe in the human breast to reach the surface of various tissues and organs and produces echo signals, collecting strong and weak signals and long and short echo times, thus forming the structure of human breast tissue image examination (16). Typical breast cancer node B ultrasound images are shown in **Figure 1**. For each image, experienced radiologists draw the ground truth ROI for cancer node detection, such as the red box in **Figure 1**.

Breast cancer node detection results are obtained by testing all BUS images through the YOLOv7 detection network (19).

**Figure 2:**
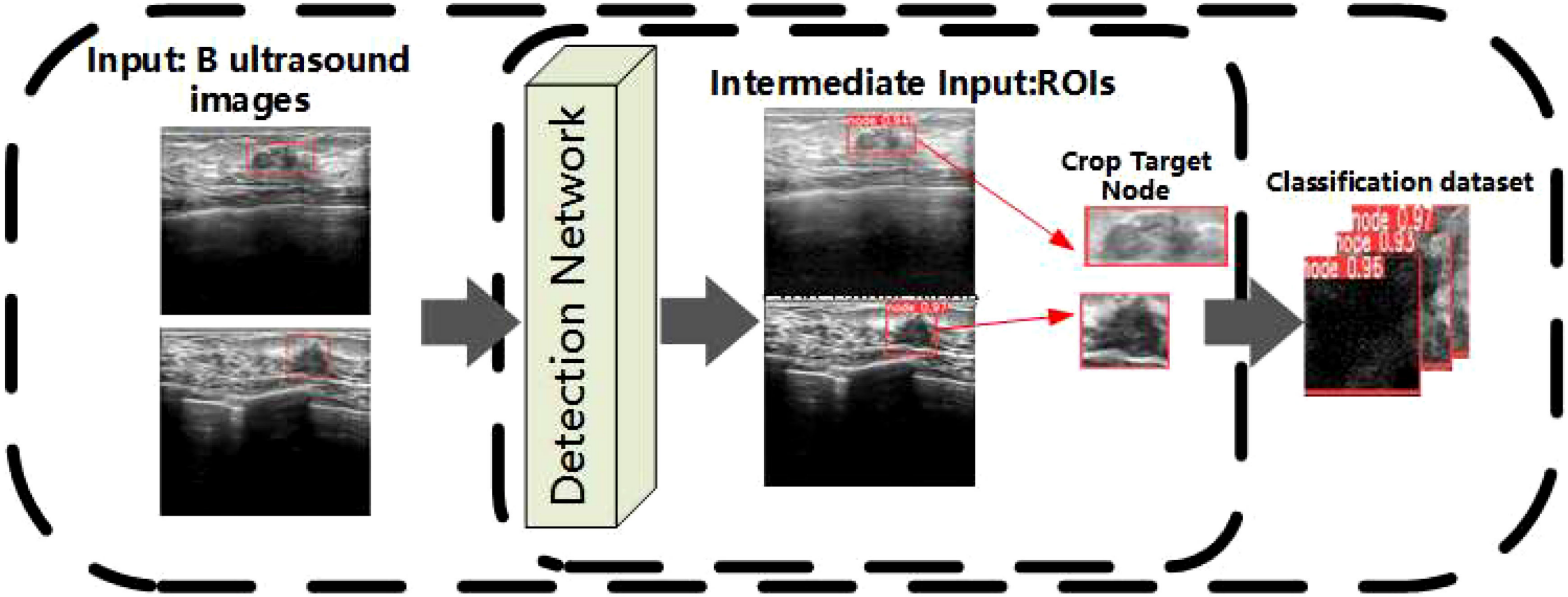
Processing stage of the classification datasets.

The specific process of inputting images into the YOLOv7 network is described as follows: First, ultrasound images of breast cancer are processed and resized to 640 × 640 pixels before being fed into the backbone network. The backbone serves as the central component of the entire detection network, initially traversing through four CBS convolutional layers composed mainly of Conv, BN, and SiLU. Following these convolutional layers, the network proceeds into the ELAN module, comprising multiple CBS convolutional layers. The input and output feature sizes remain constant, with a modification in channel number occurring after the first two convolutional layers. Subsequent input channels align with the output channels. In the final stage, there are three MP layers and the output of ELAN. The MP layers primarily consist of a blend of maximum pooling layers and convolutional layers, and the outputs of the three MP layers correspond to the outputs of C3/C4/C5. In the head section, the feature map C5 obtained from the final output of the backbone undergoes SPPCSP processing, leading to a reduction in channel count from 1,024 to 512. The SPPCSP module, building upon the SPP module, incorporates a concat operation at the end to merge it with the feature map before the SPP module. The resulting C5 is initially integrated top-down with C4 and C3, producing P3, P4, and P5. Subsequently, adopting a bottom-up approach, P4 and P5 are fused. The channel counts are adjusted through the outputs P3, P4, and P5. Lastly, a 1 × 1 convolution is applied to predict the objectness, class, and bbox components. The final breast cancer node image is obtained by clipping the result of the detection, and all breast cancer node images are used as the datasets of the classification network. The above process is shown in **Figure 2**.

For classification, after obtaining the breast cancer node datasets of breast cancer, we selected four immune indices in immunohistochemistry as the basis for judging the image of breast nodes; the four indicators are ER, PR, HER-2, and Ki-67.

When cells become cancerous, ER and PR are deleted to varying degrees. If a cell still retains ER and PR, the growth and proliferation of that breast cancer cell remain under endocrine regulation, called hormone-dependent breast cancer; if ER and PR are missing, the growth and proliferation of this breast cancer cell are no longer under endocrine regulation, and it is called hormone-independent breast cancer (20). HER-2 reflects the prognosis situation of breast cancer, which has a kinase activity and can be detected by immunohistochemistry, FISH, and so on; HER-2-positive overexpression, which can be controlled by drugs targeting its gene overexpression, can inhibit the progression of tumors (21) effectively. Immunohistochemistry of Ki-67 belongs to the common detection items in the pathology department, and it is an indicator representing the value added to the cells. A higher index indicates a higher degree of malignancy of the tumor cells, which indicates how well the tumor proliferates. Its higher value, representing the faster proliferation of tumor cells with a higher degree of malignancy, tends to simultaneously predict a greater sensitivity to chemotherapeutic agents and suitability for chemotherapy (22). Breast cancer node images were classified according to the immunohistochemical findings corresponding to the ultrasound images provided by the provincial hospital of Shandong First Medical University; the results of ER and PR are divided into two groups: hormone-dependent breast cancer and hormone-independent breast cancer. When the histochemical result is regular, hormone-dependent breast cancer is detected; when the histochemical result is negative, hormone-independent breast cancer is present. HER-2 can be divided into four types: negative and positive. Positive expression can also be divided into three expression results according to the degree of positive expression. In the immunohistochemical Ki-67 results, 14% is the boundary, less than 14% is low expression, and more than or equal to 14% is high expression. If it is more than 60%, it often indicates that the degree of malignancy is very high, most of which are triple-negative breast cancer, indicating the possibility of poor prognosis.

Based on statistical validation using the patient’s medical records, it was found that the negative and positive results of these four indicators are highly correlated with subsequent treatment and diagnosis (such as biopsy surgery). Substantial evidence is available in numerous publications. Junnan Xu has found that ER, PR, HER-2, and Ki-67 expression levels can predict the tumor mutation burden (TMB) in breast cancer patients, which is significant for prognosis and treatment decisions (23). Mustapha Abubakar et al. researched the combination of ER, PR, HER-2, and Ki-67 in chemometric analysis for assessing breast cancer images, concluding that these four indicators greatly impact the chemotherapy decisions for breast cancer patients (24). Y. Yuan et al. investigated the expression of ER, PR, HER-2, and Ki-67 in primary and metastatic breast cancer. They concluded that the expression of ER, PR, HER-2, and Ki-67 is associated with the prognosis of breast cancer patients in both primary and metastatic lesions (25).

Statistical analysis of patients in the BUS image dataset yielded the following conclusions: in terms of gender, women accounted for 100% of the patients. Regarding age distribution, 13.6% of patients were between 30 and 40, 78.4% were between 40 and 60, and 8% were over 60. According to the medical records, 79.8% of patients had a positive ER status, while 20.2% had a negative ER status. For PR, 72.2% of patients had a positive status, and 27.8% had a negative status. Regarding HER-2 expression, 16.4% had a score of 3+, 33.2% had a score of 2+, 20.2% had a score of 1+, 16% had a score of 0, and 14.2% had a score of −. Regarding Ki-67 expression, 29.8% had low expression, 57.2% had intermediate expression, and 13% had high expression.

**Figure 3:**
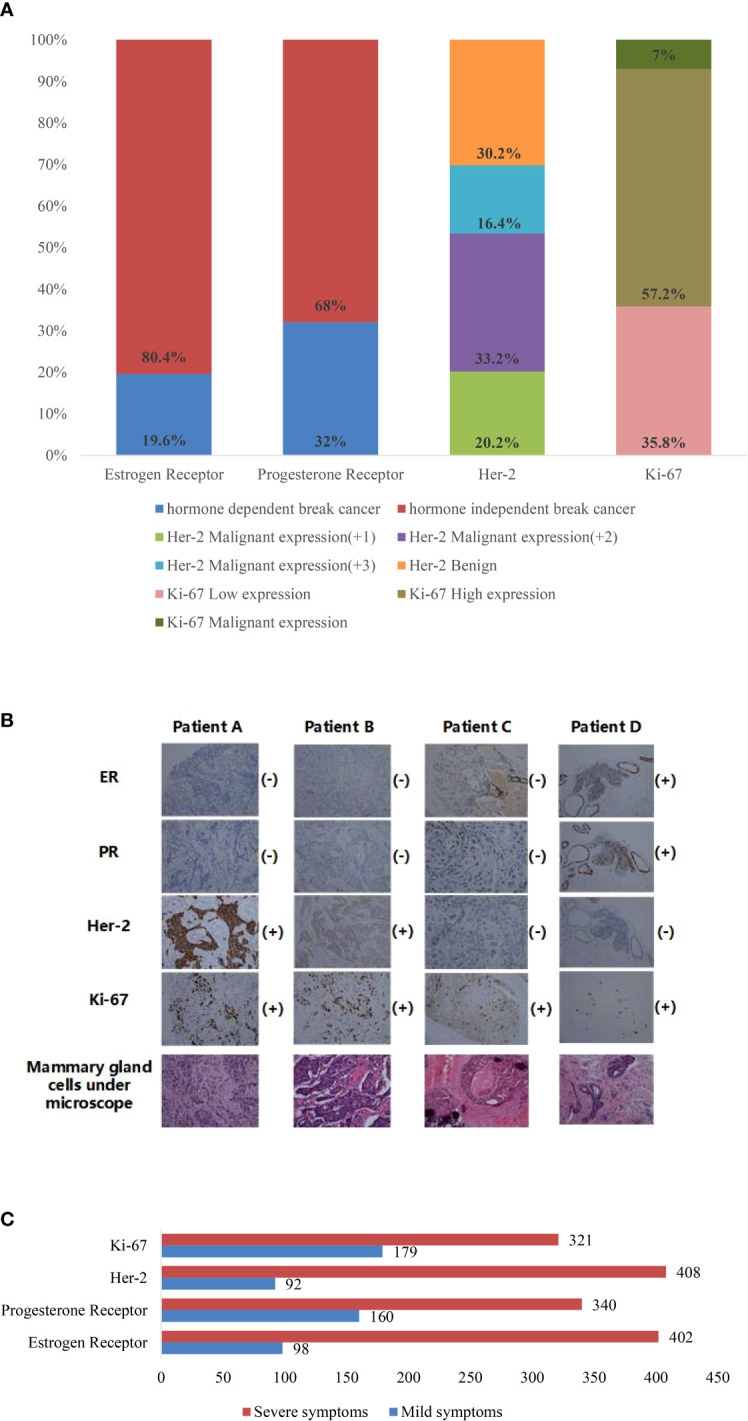
**(A)** Percentage of datasets of various immune indicators classified according to immunohistochemistry report. **(B)** Mammary cell diagram of four patients under a microscope. **(C)** The diagnosis is made according to the observation of the breast physician under the microscope, and the diagnostic results are classified into statistical figures of the datasets.

According to the above classification rules, the datasets of breast cancer nodes are divided, as shown in **Figure 3A**. It can be seen from the figure that the results of classification based only on the immunohistochemical results show that there are many classification categories, and different categories have repeatability. This is a difficult task for breast doctors to judge and test the prognosis. So, we have established a new classified dataset based on the shape, status, and activity of each breast cell observed under the microscope by a breast physician; each immune index is divided into two categories: severe (+) and mild (−). Four different patients were selected from the datasets, and the cell tissue under the microscope is shown in **Figure 3B**, while classified datasets are shown in **Figure 3C**.

## 3 Methods

### 3.1 Multistage feature distillation network

**Figure 4:**
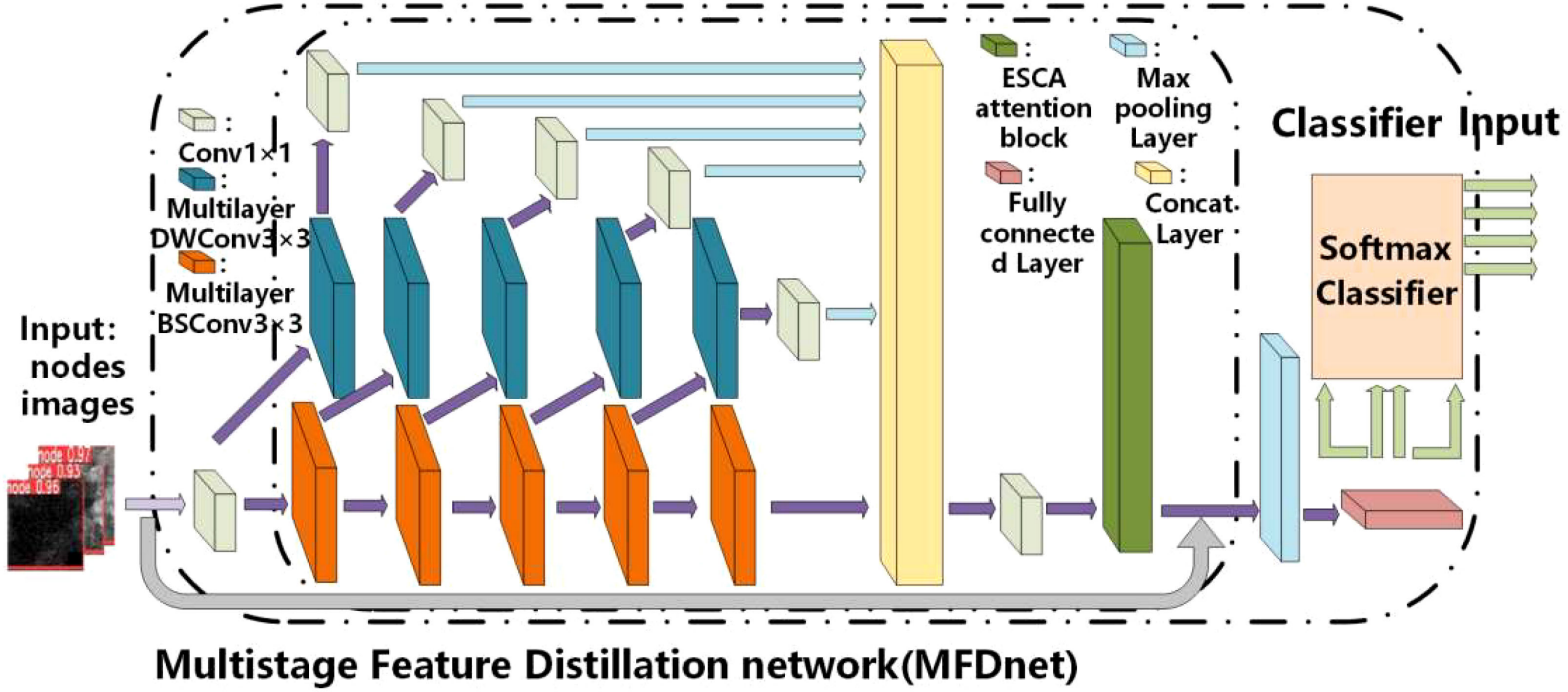
Structural diagram of the multistage feature distribution network.

The proposed overall architecture of convolutional neural networks is a multistage feature distillation network (MFD-Net), as shown in **Figure 4**, and the network architecture includes multilayer DWConv3×3, multilayer BSConv3×3, ESCA attention block, max pooling layer, fully connected layer, and soft-max classifier. MFD-Net is the backbone of the network, which is used to extract features from input images; ESCA attention block is a new attention mechanism module, which combines channel attention and spatial attention to enhance the model ability from both spatial and channel perspectives; finally, through the pooling and full connection layers, into the soft-max classifier, output classification results are derived from the four immune indicators.

The proposed CAD network is the first to apply multilabel classification to breast cancer ultrasound images, demonstrating superior performance in the image classification process. In this network, the multilevel feature distillation structure primarily performs multilevel feature extraction on input feature maps, combining the extracted multiple feature maps to extract fine-grained global features in images accurately. Depth separable convolution significantly reduces convolution parameters, lowering computational costs and improving the stability of the classification network. Including the ESCA attention module allows for capturing more detailed information about the target of interest while suppressing other irrelevant information.

After the node images are input into the multistage feature distillation network, the image passes 1×1 convolution and enters the stage of high-dimensional feature extraction. In MFD-Net, the number of feature extraction channels will be compressed in a certain proportion to form two different convolution channels, one of which enters the multilayer BSConv3×3. The convolution module forms a stacked convolution layer called the depth layer, and the other enters the multilayer DWConv3×3 convolution module. After the DWConv3×3 convolution of the feature map is followed by Conv1×1 convolutions of the feature map, making up the feature distillation process, called the distillation layer. After each subsequent multilayer BSConv3×3 convolution in the deep layer, the feature extraction channel is repeatedly compressed to form distillation layer branches. Finally, the feature extraction results of the depth layer and each distillation layer are fused. Compression and reduction of the dimension are reduced by Conv1×1 convolution to obtain the final feature extraction result as shown in Equation 1.


(1) $$Fea_{d\_1m}=DL_{1}\left(Fea_{in}\right),Fea_{d\_1}=dL_{1}\left(Fea_{d\_1m}\right),\;Fea_{depth\_1}=BL_{1}\left(Fea_{in}\right)$$

DL and dL stand for the distillation layer, which generates the features of the distillation layer, and BL stands for the depth layer, which gradually extracts the features of fine-grain size to generate the final features of the depth layer. The distillation layer was first distilled by DL and then by dL for a second distillation to obtain the final multilayer distillation characteristics. By analogy, the remaining feature extraction steps are as follows as shown in Equation 2.


(2) $$Fea_{d\_xm}=DL_{x}\left(Fea_{depth\_\left(x-1\right)}\right),Fea_{d\_x}=dL_{x}\left(Fea_{d\_xm}\right),\;Fea_{depth\_x}=BL_{x}\left(Fea_{depth\_\left(x-1\right)}\right)$$

Through the distillation features generated by the distillation layer at different stages and the feature map finally generated by the depth layer, the channel dimensions are transferred and fused. Finally, the dimension is reduced and compressed through Conv1×1 convolution as shown in Equation 3.


(3) $$Fea_{Final}=Conv\left(Concat\left(Fea_{d_{1}},Fea_{d_{2}},Fea_{d_{3}},Fea_{d_{4}},,,Fea_{d\_x},,Fea_{depth\_x}\right)\right)\;\;\;\;$$

Concat means to operate only along the channel dimension. ${\text{Fea}}_{\text{Final}}$ is a compressed feature, and  Conv (·) represents Conv1×1 convolution.

#### 3.1.1 Multilayer DWConv3×3 and multilayer BSConv3×3

**Figure 5:**
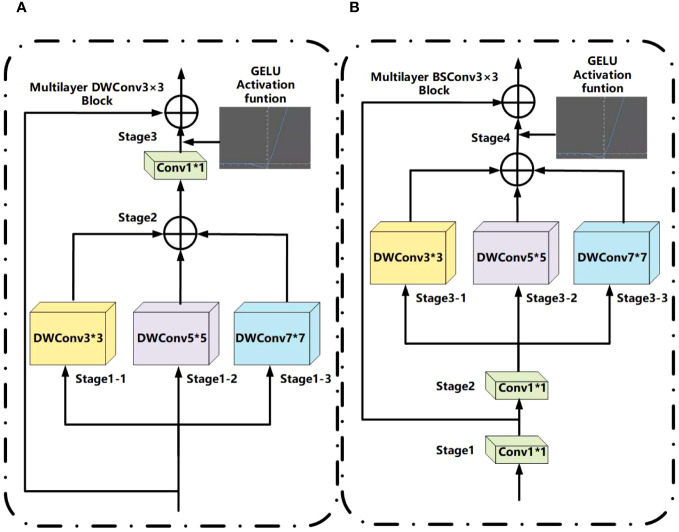
**(A)** Multilayer DWConv3×3 structure. **(B)** Multilayer BSConv3×3 structure.

As shown in **Figure 5A**, multilayer DWConv3×3 is mainly due to changes in the depthwise separable convolution (26). The DWConv3×3 structure integrates depthwise (DW) and pointwise (PW), employed for extracting feature maps during feature extraction. In contrast to conventional convolution operations, this approach reduces the number of parameters and computational costs, thereby enhancing the efficiency of feature extraction. The main change is to propose the depth convolution with different kernel sizes to form multilayer depth convolution, use three convolution kernels of different sizes on the new feature map, then combine them, and use Conv1×1 convolution for the combined feature map scales of the channel. Finally, use the residual connection to connect the input and output (27). The combination method in MFD-Net uses an additive method; its advantage is that it can extract image features of different depths and capture more information in space, and the GELU activation function is added to stabilize model feature extraction ability (28).

In **Figure 5B**, multilayer BSConv3×3 is mainly constructed according to blueprint separable convolutions (BSConv) (29). Because DWConv3×3 essentially conducts cross-kernel correlations instead of correlations within a single kernel, the BSConv3×3 structure involves swapping the order of DW and PW based on DWConv3×3. This modification enables more effective separation of standard convolutions, thereby enhancing the extraction of fine-grained features. The principle of BSConv3×3 is that the convolution kernel of deeply separable convolution will be optimized and trained using backpropagation, in which Conv1×1 convolution is first decoupled in low rank. The principle of the multilayer BSConv3×3 proposed is similar to that of multilayer DWConv3×3 above, or ordinary depth convolution is decomposed into depth convolutions of different kernel sizes and then added.

In Conv1×1 convolution, the weight *K* is highly correlated in line direction. Decomposition of *Q* not only enlarges the convolution space but also reduces the number of parameters. We did a low-rank decomposition of the weight *K* as follows as shown in Equation 4.


(4) $$K=K^{A}\times K^{B}$$

where $K^{A}$ and $K^{B}$ are low-rank decompositions of K ; following the same procedure, low-rank decompositions are again performed on $K^{A}$ and $K^{B}$ , as illustrated below as shown in Equations 5, 6.


(5) $$\;K^{A}=P\times Q^{\prime },\;K^{B}=Q^{\prime }\times Q$$

(6) $$Q^{\prime }=\left[\mu \times Q\right],\mu \in \left(0.0,1.0\right)$$

After the above rearrangement, the conventional depth separable convolution can be transformed to the following formula. By rearranging the weights $k_{Q^{\prime }}^{A}$ 1, … $\;k_{Q^{\prime }}^{A}$ *M* into the *M*×1×1 array $\tilde{\;k_{Q^{\prime }}^{A}}$ , and the weights $k_{P}^{B}$ 1, … $\;k_{P}^{B}$ *M* into the *M′*×1×1 array $\tilde{\;k_{P}^{B}}$ as shown in Equations 7, 8.


(7) $$M_{Q'}^{'}=M\times \tilde{k_{Q^{\prime }}^{A}},\;N_{P'}^{'}=M^{\prime }\times \tilde{k_{P}^{B}}$$

(8) $$N_{P'}=N_{P'}^{'}\times B^{\left(n\right)}$$

BSConv comprises three parts: i) the input tensor is projected into a *Q* dimensional subspace via a 1×1 pointwise convolution with kernels $\;k_{Q^{\prime }}^{A}$ 1,… $\;k_{Q^{\prime }}^{A}$ *M*. ii) Another 1×1 pointwise convolution with kernels $k_{P}^{B}$ 1,… $k_{P}^{B}$ *M* is applied to the result of the first step. iii) A *K*×*K* depthwise convolution with kernels $B^{\left(1\right)}$ ,… $B^{\left(n\right)}$ is applied to the result of step 2.

We extend the image to the space range, where *k* represents the convolution kernel depth; suppose the input tensor size is $h\times \omega \times d_{i}$ , the output tensor size $h\times \omega \times d_{j}$ , so their calculation formula is $h\times \omega \times d_{i}\times d_{j}\times k$ , the input channels are *M*, and the following formulae can be obtained as shown in Equations 9–12.


(9) $$N_{P'}^{'}=h\times \omega \times d_{i}\times \sum_{m=0}^{M}k_{m}^{2},K=\left\{k_{1},k_{2},\ldots k_{m}\right\}$$

(10) $$N_{P'}=h\times \omega \times d_{i}\times \sum_{m=0}^{M}k_{m}^{2}\times B^{\left(n\right)},K=\left\{k_{1},k_{2},\ldots k_{m}\right\}$$

(11) $$N_{P'}=\left(M-1\right)\times h\times \omega \times d_{i}\times d_{j}\times \sum_{m=0}^{M}k_{m}^{2}\times B^{\left(n\right)},K=\left\{k_{1},k_{2},\ldots k_{m}\right\}$$

(12) $$N_{P'}=h\times \omega \times d_{i}\times (d_{j}\times M+\sum_{m=0}^{M}k_{m}^{2})\times B^{\left(n\right)},K=\left\{k_{1},k_{2},\ldots k_{m}\right\}$$

The above formulae mainly outline the convolution process in **Figure 5B**, decompose ordinary depth separable convolutions into separable convolutions with multiple different depths, and finally output the characteristic graph through residual connection, and the specific steps are as follows: 1×1 convolution + 1×1 convolution + (3×3 convolution + 4×4 convolution + 6×6 convolution).

#### 3.1.2 ESCA attention block

With the wide application of the human attention mechanism, the visual attention mechanism is gradually popularized in neural networks, such as the squeeze-and-excitation (SE) module (30) and coordinate attention (CA) (31), which forces the adopted model to pay more attention to the discriminative features of the objects to improve its recording performance.

**Figure 6:**
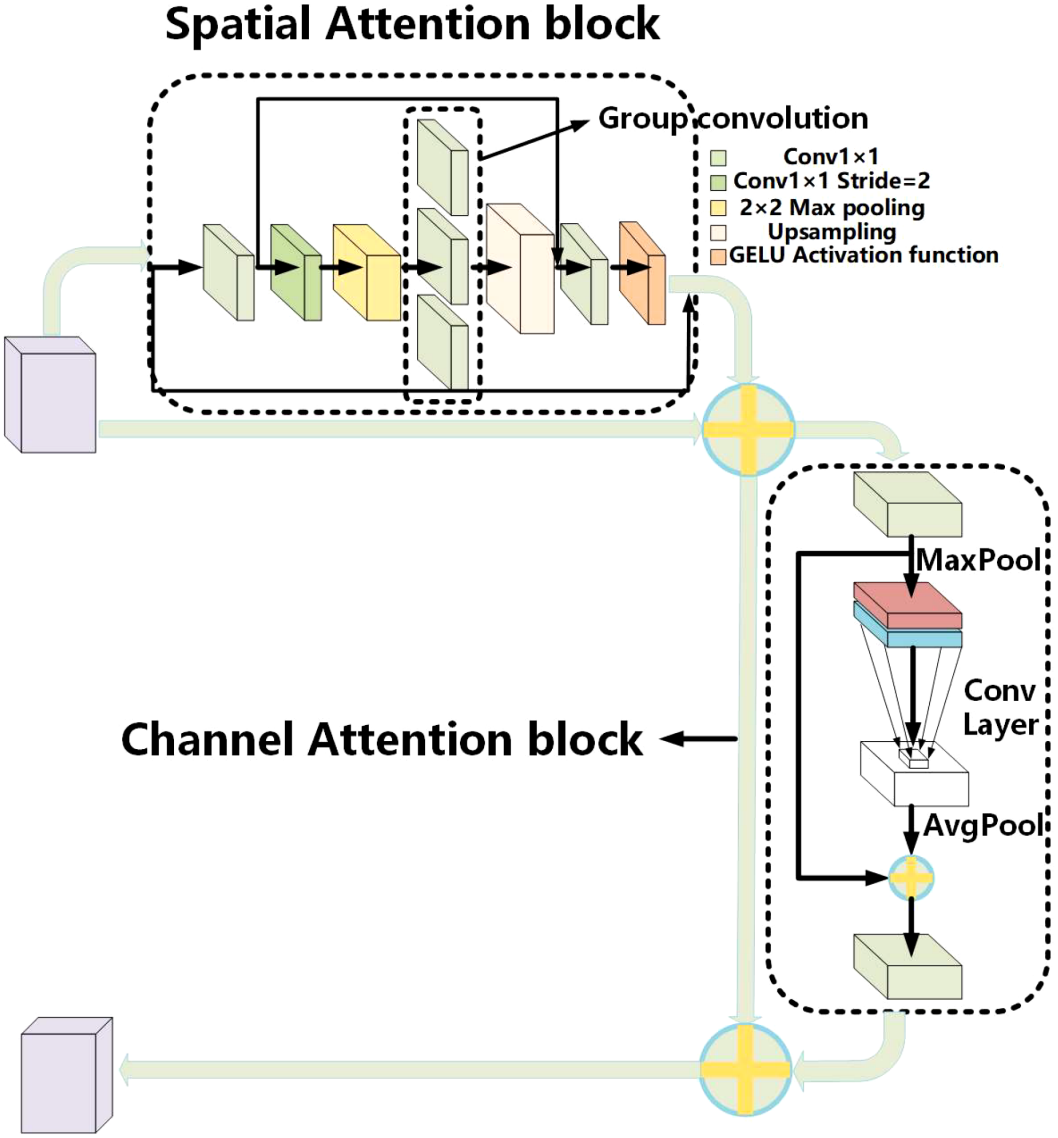
Overall structure diagram of the ESCA attention mechanism.

Based on the construction idea of CBAM (32), the ESCA attention module is proposed. ESCA has two independent submodules, the channel attention module (CAM) and spatial attention module (SAM), which can improve the model classification ability in channel and space. These steps are as follows: paying attention to the input feature map on the spatial first, then paying attention to the channel, combining them in series, and finally output the feature map. The overall structure is shown in **Figure 6**.

The main purpose of the spatial attention module is to achieve a more comprehensive and deeper receptive field range. First, use Conv1x1 convolution to reduce the number of channels on the input feature map. Then, use the convolution with 2×2 max pooling and Conv to expand the receptive field range. Then, use upsampling to obtain the features of the input size. Add a residual connection to ensure that the output image retains the original features. Finally, using Conv1x1 convolution and sigmoid functions, the result obtained is a dot multiplied by the input to obtain the output characteristics of the spatial attention module.

The channel attention module is mainly inspired by ECA attention (33). The main purpose is to enhance the channel characteristics of the input feature map, reduce parameter calculation, and enhance the model’s accuracy. First, the input feature map on the spatial dimension is pooled on a global average to achieve spatial feature compression. Then, the compressed spatial feature map learns channel characteristics by Conv1×1 convolution. Finally, the channel attention feature map 1×1×C and the original input feature map *H*×*W*×*C* are multiplied channel by channel to output the feature map with channel attention.

### 3.2 Loss function

As this project carries out multilabel classification for immunohistochemical tissues, soft-max is the most popular multiclassification classifier in recent years, and it can increase or decrease the signal exponentially, highlighting the output results to be enhanced (34). Therefore, the output layer often adds soft-max as a classifier to complete the multiclassification purpose. The output results take the cross-entropy loss in the loss function selection to evaluate the distribution difference between the real label and the predicted value (35). The cross-entropy loss function is expressed as follows as shown in Equations 13, 14:


(13) $$\;L=-\sum_{i=1}^{C}y_{i}\log\left(\sigma_{i}\right)$$

(14) $$\sigma \left(y=i\right)=\frac{e^{Zi}}{\sum_{j=0}^{C}e^{Zj}},i\in \left\{0,\ldots ,C\right\}$$

*C* is the category of immunohistochemical cells or the total number of labels, *y_i_* is the sample’s true label and the ROI box’s label, >0, and =1, $\sigma_{i}$ represents the probability that sample *i* is predicted to be a positive class. Inspired by the concurrent soft-max, the gradient descent formula adds concurrent soft-max to two weighting coefficients (18) as shown in Equation 15.


(15) $$w_{t+1}=w_{t}-\gamma \frac{\delta L}{\delta w},$$

where *L* is the cross-entropy loss function, and *w* is the weight parameter. This loss function was applied to the output layer, as shown in Equations 16, 17.


(16) $$L=-\sum_{i=1}^{C}y_{i}\log\left(\sigma_{i}^{*}\right)$$

(17) $$\sigma^{*}\left(y=i\right)=\frac{e^{Zi}}{\sum_{j=0}^{C}\left(1-y_{i}\right)\left(1-r_{ij}\right)e^{Zj}+e^{Z_{i}}},i\in \left\{0,\ldots ,C\right\}$$

where $r_{ij}$ is the probability of the simultaneous occurrence of *tag i* and *tag j* obtained through the advanced statistics of the training set; others still use the soft-max classifier to calculate the output results.

## 4 Results

The experimental platform was the Ubuntu 18.04 LTS operating system. The experimental environment included Python 3.8, CUDA 10.0, and PyTorch 1.10.0. The accelerator was an NVIDIA GeForce GTX TITAN X graphical processing unit.

The standard evaluation criteria were used to evaluate the performance of the multistage feature distillation network. They included precision (PREC), recall (REC), accuracy (ACC), and F1 score (F1), which were defined as follows:


$$\text{PREC}=\frac{\text{TP}}{\text{TP}+\text{FP}},\text{ REC}=\frac{\text{TP}}{\text{TP}+\text{FN}},\text{ F}1=2\times \frac{\text{PREC}\times \text{REC}}{\text{PREC}+\text{REC}},\text{ ACC}=\frac{\text{TP}+\text{TN}}{\text{TP}+\text{TN}+\text{FP}+\text{FN}}$$

where TP, FP, TN, and FN represent true positives, false positives, true negatives, and false negatives, respectively.

The dataset used for the entire experiment is a breast cancer ultrasound image dataset curated by ourselves. The process of experimenting with the classification network mainly involves the following steps: firstly, organizing the breast cancer ultrasound dataset; secondly, defining the network structure; next, training the defined network model; then, testing the network model; and finally, predicting breast cancer ultrasound images. The breast cancer ultrasound image dataset consists of ultrasound images from 294 patients, totaling 10,000 images.

### 4.1 Model contrast experiments

First, the MFD-Net was trained and verified on the breast cancer node datasets, divided into a 70% training set and 30% verification set, and selected batch size of 8 and training epoch of 200. The four selected immunohistochemical indicators ER, PR, HER-2, and Ki-67, respectively, output precision, recall, accuracy, and F1, then their performance was compared with those of the 10 most popular classification models in recent years, namely, ShuffleNetV2 (36), EfficientNet (37), MLP-Mixer (38), Twins (39), PoolFormer (40), VAN (41), T2T-ViT (42), Swin Transformer v2 (43), MobileNetV3 (44), and RepVGG (45). For example, **Table 1** shows the precision, recall, accuracy, and F1 score of ER output of various models on the same validation datasets.

**Table 1:** ER immune index taken as an example for classifying the performance of different structures.

Model	Precision	Recall	Accuracy	F1
ShuffleNetV2	88.64%	75.25%	82.58%	81.39%
EfficientNet	87.24%	73.12%	81.54%	79.55%
MLP-Mixer	76.67%	65.25%	80.42%	70.50%
Twins	80.43%	68.75%	80.56%	74.13%
PoolFormer	73.88%	69.24%	82.65%	71.48%
VAN	87.24%	73.12%	81.50%	81.07%
T2T-ViT	88.64%	72.25%	82.58%	79.61%
Swin Transformer v2	88.66%	70.36%	82.78%	78.45%
MobileNetV3	86.79%	70.88%	83.62%	78.03%
RepVGG	75.95%	71.88%	82.46%	73.85%
**MFD-Net**	**89.33**%	**75.63**%	**83.86**%	**81.91**%

Since the four immune indicators of the network are output simultaneously, to make the advantages of the proposed classification network more obvious, the four immune indicators are extracted and compared with 10 different classification networks, taking the ER immune index as an example. **Table 2** shows the PR immune index. **Table 3** shows the HER-2 immunity index, and **Table 4** shows the Ki-67 immunity index.

**Figure 7:**
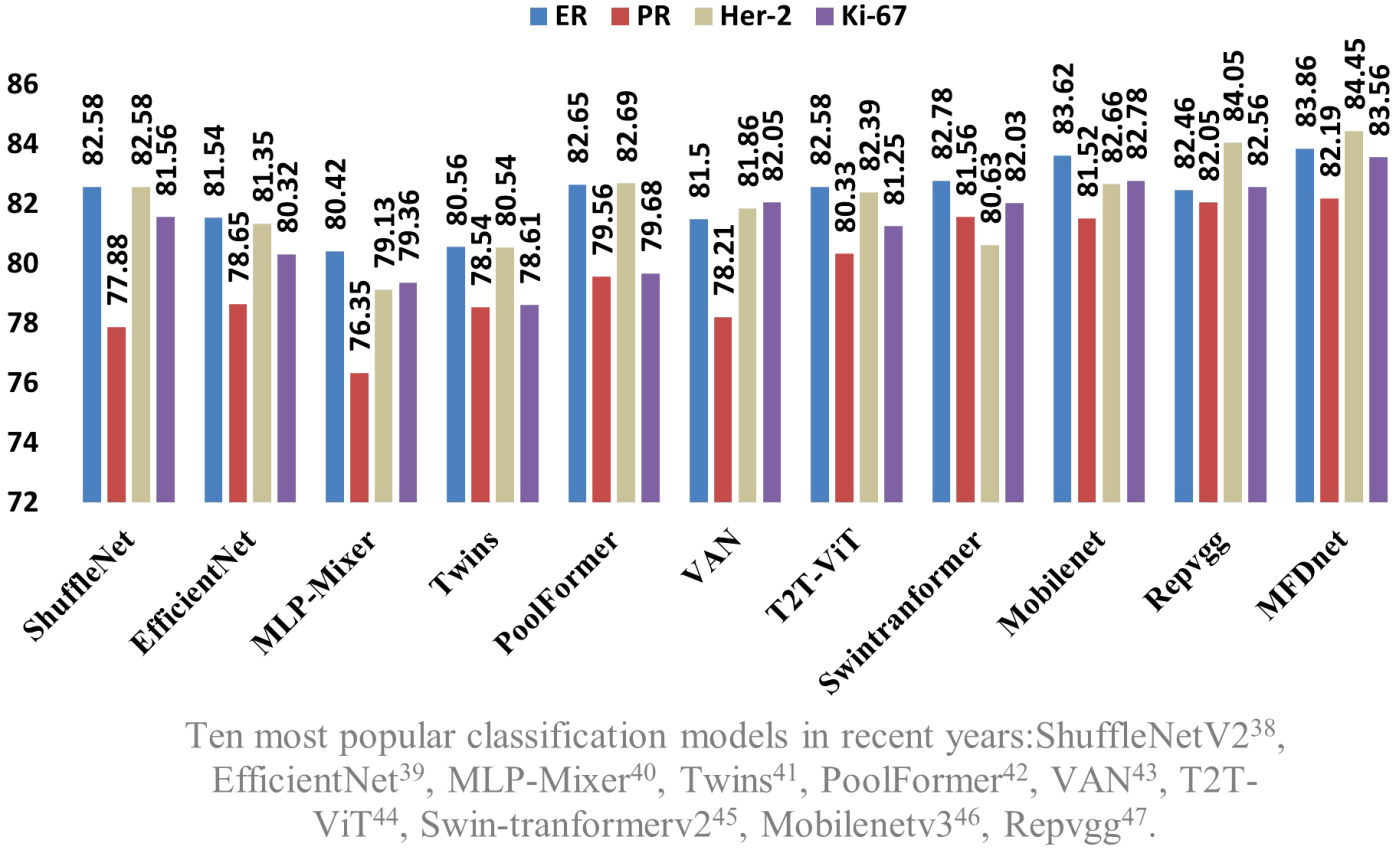
Comparison of the accuracy of the proposed network model and the SOTA models in four immune indicators.

The results obtained by the proposed method were compared with those predicted by other 10 state-of-the-art neural networks as shown in **Tables 1**–**4** where bold values represent the training results of the proposed model MFD Net, emphasizing that all training results are superior to other models. Thus, the proposed classification network outperformed all 10 state-of-the-art networks in accuracy, precision, recall, and F1. **Figure 7** shows the accuracy comparison of the four immune indicators in different network models. It can be seen from the figure that the accuracy of the network model proposed in this paper is better than the popular SOTA models in the comparative experiment.

**Table 2:** PR immune index taken as an example for classifying the performance of different structures.

Model	Precision	Recall	Accuracy	F1
ShuffleNetV2	87.39%	70.05%	77.88%	80.75%
EfficientNet	86.78%	70.44%	78.65%	77.76%
MLP-Mixer	84.36%	67.25%	76.35%	74.84%
Twins	82.56%	68.36%	78.54%	74.79%
PoolFormer	82.78%	67.37%	79.56%	74.28%
VAN	84.28%	68.78%	78.21%	75.74%
T2T-ViT	86.30%	70.36%	80.33%	77.51%
Swin Transformer V2	85.44%	68.35%	81.56%	75.94%
MobileNetV3	83.65%	69.58%	81.52%	75.96%
RepVGG	85.85%	71.78%	82.05%	78.18%
**MFD-Net**	**88.36**%	**75.53**%	**82.19**%	**81.44**%

**Table 3:** HER-2 immune index taken as an example for classifying the performance of different structures.

Model	Precision	Recall	Accuracy	F1
ShuffleNetV2	78.69%	68.01%	82.58%	72.96%
EfficientNet	78.50%	67.53%	81.35%	72.60%
MLP-Mixer	77.58%	68.56%	79.13%	72.79%
Twins	76.89%	67.25%	80.54%	71.74%
PoolFormer	78.59%	67.66%	82.69%	72.76%
VAN	77.25%	68.15%	81.86%	72.41%
T2T-ViT	80.33%	68.63%	82.39%	74.02%
Swin Transformer V2	79.67%	70.05%	80.63%	74.55%
MobileNetV3	80.66%	70.47%	82.66%	75.22%
RepVGG	78.89%	72.56%	84.05%	75.59%
**MFD-Net**	**82.36**%	**75.32**%	**84.45**%	**79.61**%

**Table 4:** Ki-67 immune index taken as an example for classifying the performance of different structures.

Model	Precision	Recall	Accuracy	F1
ShuffleNetV2	81.36%	74.97%	81.56%	78.18%
EfficientNet	82.87%	74.77%	80.32%	78.61%
MLP-Mixer	80.24%	68.17%	79.36%	73.71%
Twins	79.58%	67.81%	78.61%	73.22%
PoolFormer	80.89%	69.12%	79.68%	74.54%
VAN	81.78%	69.23%	82.05%	74.98%
T2T-ViT	81.55%	70.56%	81.25%	75.65%
Swin Transformer V2	82.36%	73.48%	82.03%	77.66%
MobileNetV3	84.56%	74.03%	82.78%	78.94%
RepVGG	83.54%	72.36%	82.56%	77.54%
**MFD-Net**	**85.33**%	**77.33**%	**83.56**%	**81.13**%

Furthermore, in comparison with deep learning methods, the proposed network structure is also compared with the network structure used in the articles on breast cancer published in international academic conferences or journals in the past 3 years. Since these articles are limited to single immunological markers, they are binary classified according to immunohistochemical results. Among them, Kalafi et al. carried out experiments on invasive ductal carcinoma types of malignant lesions and fibroadenoma types of benign lesions in the improved VGG network in BUS images in 2021 (14). Rasaee and Rivaz also classified benign and malignant breast nodules in BUS images in 2021 and carried out classification experiments on them through the new network of improved classification of ResNet-50 (46). In 2022, Gheflati and Rivaz used different enhancement strategies to classify BUS images through Vision Transformer (ViT) for the first time (47). Muhammad et al. developed an end-to-end integrated pipeline image classification for BUS, using the pretrained VGG16 and the closely connected neural network learning method for experiments (48). The MFD-Net network was modified into a binary classification network, and the network structure for the classification of BUS images was designed. After that, a comparative experiment was carried out with the ER immune index as an example. The experimental results are listed in **Table 5**.

**Table 5:** ER immune index taken as an example for classifying the performance of network structure proposed by popular articles in the last 3 years.

Method	Precision	Recall	Accuracy	F1
Kalafi et al. [16]	90.93%	72.5%	81.67%	80.67%
Rasaee and Rivaz [48]	86.63%	74.12%	78.54%	79.88%
Gheflati and Rivaz [49]	85.45%	75.2%	80.42%	80.01%
Muhammad et al. [50]	88.15%	76.3%	80.62%	81.84%
**This study**	**89.33**%	**75.6**%	**83.56**%	**81.91**%

It can be seen from **Table 6** that the proposed network structure is superior to three models in precision, three models in recall, and four models in accuracy and F1. The proposed network’s performance has been the best among the binary classification networks for BUS image classification in the past 2 years.

**Table 6:** The ER immune index taken as an example for the ablation experiment of the multilayer deep separation convolution module in MFD-Net.

Method	Precision	Recall	Accuracy	F1
DWConv3×3, BSConv3×3	87.54%	75.02%	82.17%	80.79%
Multilayer DWConv3×3, multilayer BSConv3×3	89.33%	75.63%	83.56%	81.91%

### 4.2 Module contrast experiment

In the MFD-Net proposed for the first time, multilayer depth separable convolution and multilayer reverse depth separable convolution are used in the classification network. Compared with ordinary depth separable convolution and reverse depth separable convolution, more feature maps can be extracted, more feature information can be captured, and the deep separable convolution and multilayer depth separable convolution are added to MFD-Net to experiment under the same conditions of other factors. The results are summarized in **Table 6**.

It can be seen from **Table 6** that the multilayer depth separable convolution can effectively improve each index in the classification tasks.

### 4.3 Ablation experiment

Ablation experiments were conducted on the breast nodule datasets to further study the contribution of each component of MFD-Net to its performance. The essence of the ablation experiment is to highlight the advantages of innovation points in the model design process and to ensure improvement of innovation points in training or testing other datasets. In the MFD-Net network, the most important innovation point is to use the structure of characteristic multistage distillation to design the overall network backbone and carry out three characteristic distillations of different modules on the input characteristic map; the feature distillation module determines the feature extraction effect of fine-grained images. To further show the effect of feature distillation structure used in fine-grained images, three different feature distillation modules were ablated, and the results are shown in **Table 7** below.

**Table 7:** The ER immune index taken as an example for the ablation experiment of the distillation module in MFD-Net.

Method	Model (MFD-Net)		
Multilayer BSConv3×3	√	√	√
Multilayer DWConv3×3	√	√	
Conv1×1	√		
**Accuracy**	**83.56**%	**83.36**%	**82.45**%

Each distillation module of the designed distillation network has played a role in the classification of fine-grained images, and the higher the distillation level, the higher the accuracy. Multilayer BSConv3×3 forms the basic backbone of MFD-Net, making the accuracy of the network reach the same level as that of the SOTA algorithm in recent years; on this basis, multilayer DWConv3×3 is added. The secondary feature extraction of the feature map greatly improved the accuracy; the reason for adding *c* to the final distillation layer was to reduce the dimension of the overall feature map and also to improve the classification network’s accuracy slightly. Therefore, the multilevel feature distillation structure enhanced the accuracy of the classification network.

The ESCA attention module has shown good performance in improving the accuracy of the MFD-Net network. It was based on the construction idea of the CBAM attention module, which combined spatial attention with channel attention to improve the classification ability of the model. Both of the attentions affected the accuracy value. The ablation experiment of the ESCA attention module was carried out with the same other factors, and the results are listed in **Table 8**.

**Table 8:** The ER immune index taken as an example for the ablation experiment of the attention module in MFD-Net.

Method	Model (MFD-Net)		
With no attention module			√
ESCA attention without channel attention		√	
ESCA attention	√		
**Accuracy**	**83.56**%	**83.14**%	**82.55**%

As can be deduced from **Table 8**, adding an ESCA attention module improved the accuracy by one percentage point compared with the network without this module. Thus, adding the ESCA attention module to the MFD-Net improved the accuracy, with both channel and spatial attention submodules contributing to this enhancement.

## 5 Conclusions and limitations

This study presents a deep learning model to classify BUS images based on early immunohistochemical results of breast cancer patients, which is mainly used for the predictive treatment and diagnosis of breast cancer patients. For the first time, this model inputs four immunohistochemical results into the classification network simultaneously and outputs four immunohistochemical classification results simultaneously, thus realizing the multiclassification of mammary ultrasound images. Moreover, this network model has performed better than the advanced classification network in recent years. This CAD method is a reliable second opinion for seasoned radiologists and a valuable resource for junior ones. In the future development of medical imaging, this CAD method can be integrated with radiologists’ experience and domain knowledge, enhancing clinical relevance.

This study proposes a multistage feature distillation network structure, and it has been applied to image classification for the first time with good results. In addition, depthwise separable convolution and reverse depthwise separable convolution are applied to distillation networks, increasing different convolution core depths to multilayer depthwise separable convolution and multilayer reverse depthwise separable convolution, which showed good performance in classification tasks. A new attention mechanism is designed in the proposed network structure and applied to immunohistochemical classification in ultrasound images to allow the model to learn more important texture information. Comparing the accuracy of the proposed network with that of several advanced classification networks, it is proven that the proposed model is superior to existing algorithms in immunohistochemical classification of ultrasound images and can achieve the effect of simultaneous classification of multiple immune indices, which is a breakthrough in the whole breast cancer image processing field.

The proposed CAD implementation can alleviate several medical diagnostic problems. First, the diagnostic results of the same ultrasound image by different radiologists may be influenced by human factors. Applying quantitative criteria in CAD methods ensures accurate and consistent results, which may remove barriers to observer differences (49). Secondly, the CAD method has good diagnostic performance and can be used as an assistant tool to help radiologists diagnose breast cancer clinically (50). According to the immunohistochemical report sheet, the correct diagnosis direction can be made in the future to prevent the occurrence of late symptoms of breast cancer (51). Finally, the results of immunohistochemical classification by CAD can greatly reduce the manpower and resources required in the later treatment of breast cancer and improve the efficiency of physicians (52).

However, this study has several limitations. First, due to time constraints, relatively few BUS images and corresponding immunohistochemical reports are collected herein. Second, the experiments were only performed on the BUS datasets from the provincial hospital of the first medical university in Shandong, China, and no validation was performed on other datasets. Therefore, there may be a systematic bias in the results. Third, breast cancer immunohistochemical results are derived by physicians through a variety of techniques, so the results are highly dependent on the physician’s experience.

In conclusion, a deep learning-based CAD framework guided by BUS images as a dataset and immunohistochemical results analysis is proposed to design a novel multilevel feature distilled classification network (MFD-Net) for the immunohistochemical classification of BUS images. This study is the first to apply multiple immunohistochemical classifications to BUS images. The proposed method outperforms the classification networks in recent years in classification accuracy and the classification network applied in a breast image article in the last 2 years. Utilizing the CAD model proposed in this study notably improves the efficiency of identifying fine-grained medical images. Additionally, it effectively addresses the challenge of multilabel recognition in medical imaging, assisting radiologists in the multilabel identification of medical images. The proposed CAD method can serve as a reliable second opinion for radiologists, helping them to avoid misdiagnosis due to work overload. In addition, it can provide useful advice to junior radiologists with limited clinical experience. Future studies can consider adding the radiologists’ experience and domain knowledge to the deep learning-based CAD approach to make it more clinically meaningful.

## Data availability statement

The raw data supporting the conclusions of this article will be made available by the authors, without undue reservation.

## Ethics statement

Ethical approval was not required for the studies involving humans because This study does not involve ethics approval. The studies were conducted in accordance with the local legislation and institutional requirements. The participants provided their written informed consent to participate in this study. Written informed consent was obtained from the individual(s) for the publication of any potentially identifiable images or data included in this article.

## Author contributions

ZZ: Project administration, Supervision, Writing – original draft, Writing – review & editing. DY: Conceptualization, Methodology, Validation, Writing – original draft, Writing – review & editing. JQ: Data curation, Investigation, Writing – review & editing. LS: Data curation, Investigation, Writing – review & editing. QW: Data curation, Investigation, Writing – review & editing. HZ: Data curation, Investigation, Writing – review & editing. JD: Conceptualization, Methodology, Writing – review & editing.
